# Supplementary material for: Comparative survival analyses among captive chimpanzees (Pan troglodytes) in America and Japan
Source: PeerJ. 2021 Aug 12;9:e11913. doi: 10.7717/peerj.11913 (PMC8364750; doi:10.7717/peerj.11913)
Supplement: Supplemental Information 2 — In these life tables (Tables S1 and S2), nx (qx) = number of individuals at risk for mortality calculations, dx = probability of death between age x and x + 1 calculated as l(x + 1) − l(x), qx = probability of death between age x and x + 1 calculated as the number of animals that die during an age class divided by the number of animals at risk, lx (0y) = probability of survival from birth to age x, lx (1y) = probability of survival from 1 year to age x, ex = remaining life expectancy (in years) at age x, nx (mx) = number of individuals at risk for fecundity calculations, mx = fecundity or the average number of same-sex young born to individuals in that age class. [file peerj-09-11913-s002.docx]

|  | Females |  |  |  |  |  |  |  | Males |  |  |  |  |  |  |  |
| --- | --- | --- | --- | --- | --- | --- | --- | --- | --- | --- | --- | --- | --- | --- | --- | --- |
| Age (years) | nx (qx) | dx | qx | lx (0yr) | lx (1yr) | ex | nx (mx) | mx | nx (qx) | dx | qx | lx (0yr) | lx (1yr) | ex | nx (mx) | mx |
| 0 | 179.4 | 0.15 | 0.15 | 1.00 |  | 32.9 | 179.4 | 0.00 | 151 | 0.18 | 0.18 | 1.00 |  | 27.1 | 151 | 0.00 |
| 1 | 169.9 | 0.05 | 0.06 | 0.85 | 1.00 | 37.6 | 169.9 | 0.00 | 137.6 | 0.02 | 0.02 | 0.82 | 1.00 | 31.7 | 137.6 | 0.00 |
| 2 | 167.2 | 0.01 | 0.02 | 0.80 | 0.94 | 38.8 | 167.2 | 0.00 | 134.6 | 0.01 | 0.02 | 0.80 | 0.98 | 31.4 | 134.6 | 0.00 |
| 3 | 168.6 | 0.00 | 0.01 | 0.79 | 0.93 | 38.5 | 168.6 | 0.00 | 138 | 0.01 | 0.01 | 0.79 | 0.96 | 30.8 | 138 | 0.00 |
| 4 | 166 | 0.00 | 0.01 | 0.78 | 0.92 | 37.8 | 166 | 0.00 | 135 | 0.01 | 0.01 | 0.78 | 0.95 | 30.3 | 135 | 0.00 |
| 5 | 170.8 | 0.01 | 0.02 | 0.78 | 0.91 | 37.0 | 170.8 | 0.00 | 134.3 | 0.01 | 0.01 | 0.78 | 0.94 | 29.5 | 134.3 | 0.00 |
| 6 | 170.8 | 0.01 | 0.01 | 0.76 | 0.90 | 36.6 | 170.8 | 0.00 | 131 | 0.00 | 0.00 | 0.77 | 0.94 | 28.7 | 131 | 0.01 |
| 7 | 169 | 0.01 | 0.01 | 0.75 | 0.89 | 36.1 | 169 | 0.02 | 127.2 | 0.04 | 0.05 | 0.77 | 0.94 | 27.7 | 127.2 | 0.06 |
| 8 | 170 | 0.00 | 0.00 | 0.74 | 0.88 | 35.5 | 170 | 0.04 | 122.9 | 0.03 | 0.04 | 0.73 | 0.89 | 28.2 | 122.9 | 0.06 |
| 9 | 168.4 | 0.00 | 0.00 | 0.74 | 0.88 | 34.5 | 168.4 | 0.04 | 120.6 | 0.01 | 0.02 | 0.70 | 0.85 | 28.3 | 120.6 | 0.10 |
| 10 | 175.1 | 0.01 | 0.01 | 0.74 | 0.88 | 33.5 | 175.1 | 0.07 | 117.8 | 0.01 | 0.02 | 0.69 | 0.84 | 27.8 | 117.8 | 0.12 |
| 11 | 179.2 | 0.01 | 0.02 | 0.74 | 0.87 | 32.9 | 179.2 | 0.05 | 113.5 | 0.00 | 0.00 | 0.68 | 0.82 | 27.2 | 113.5 | 0.08 |
| 12 | 184.9 | 0.00 | 0.01 | 0.72 | 0.85 | 32.4 | 184.9 | 0.07 | 115.9 | 0.01 | 0.02 | 0.68 | 0.82 | 26.2 | 115.9 | 0.11 |
| 13 | 184.5 | 0.00 | 0.01 | 0.72 | 0.85 | 31.5 | 184.5 | 0.06 | 116.4 | 0.01 | 0.02 | 0.67 | 0.81 | 25.6 | 116.4 | 0.08 |
| 14 | 180.9 | 0.01 | 0.02 | 0.72 | 0.85 | 30.7 | 180.9 | 0.04 | 116.5 | 0.00 | 0.00 | 0.65 | 0.80 | 25.1 | 116.5 | 0.07 |
| 15 | 178.4 | 0.01 | 0.01 | 0.71 | 0.83 | 30.2 | 178.4 | 0.05 | 114 | 0.03 | 0.05 | 0.65 | 0.80 | 24.1 | 114 | 0.08 |
| 16 | 177 | 0.02 | 0.03 | 0.70 | 0.82 | 29.5 | 177 | 0.05 | 116.3 | 0.00 | 0.00 | 0.62 | 0.76 | 24.3 | 116.3 | 0.05 |
| 17 | 173.2 | 0.01 | 0.01 | 0.68 | 0.80 | 29.3 | 173.2 | 0.04 | 117.9 | 0.02 | 0.03 | 0.62 | 0.76 | 23.3 | 117.9 | 0.11 |
| 18 | 173.5 | 0.00 | 0.00 | 0.67 | 0.79 | 28.7 | 173.5 | 0.03 | 111.9 | 0.01 | 0.01 | 0.60 | 0.73 | 23.1 | 111.9 | 0.08 |
| 19 | 172.6 | 0.01 | 0.02 | 0.67 | 0.79 | 27.7 | 172.6 | 0.05 | 110.3 | 0.00 | 0.00 | 0.60 | 0.72 | 22.3 | 110.3 | 0.08 |
| 20 | 164.9 | 0.00 | 0.00 | 0.66 | 0.78 | 27.1 | 164.9 | 0.03 | 108.7 | 0.03 | 0.05 | 0.60 | 0.72 | 21.3 | 108.7 | 0.09 |
| 21 | 160.8 | 0.01 | 0.02 | 0.66 | 0.78 | 26.1 | 160.8 | 0.03 | 100.8 | 0.02 | 0.03 | 0.57 | 0.69 | 21.2 | 100.8 | 0.08 |
| 22 | 156.9 | 0.00 | 0.01 | 0.65 | 0.76 | 25.6 | 156.9 | 0.06 | 97.2 | 0.00 | 0.00 | 0.55 | 0.67 | 20.8 | 97.2 | 0.04 |
| 23 | 152.6 | 0.01 | 0.02 | 0.64 | 0.76 | 24.7 | 152.6 | 0.05 | 95.4 | 0.01 | 0.02 | 0.55 | 0.67 | 19.8 | 95.4 | 0.11 |
| 24 | 151.3 | 0.00 | 0.01 | 0.63 | 0.74 | 24.2 | 151.3 | 0.03 | 89.8 | 0.01 | 0.01 | 0.54 | 0.66 | 19.2 | 89.8 | 0.05 |
| 25 | 148.5 | 0.01 | 0.01 | 0.63 | 0.74 | 23.4 | 148.5 | 0.05 | 86.7 | 0.01 | 0.01 | 0.53 | 0.65 | 18.4 | 86.7 | 0.05 |
| 26 | 145 | 0.01 | 0.01 | 0.62 | 0.73 | 22.6 | 145 | 0.05 | 84.3 | 0.01 | 0.01 | 0.53 | 0.64 | 17.6 | 84.3 | 0.08 |
| 27 | 140.8 | 0.01 | 0.02 | 0.61 | 0.72 | 22.0 | 140.8 | 0.02 | 81.2 | 0.01 | 0.02 | 0.52 | 0.64 | 16.8 | 81.2 | 0.03 |
| 28 | 135.4 | 0.01 | 0.02 | 0.60 | 0.70 | 21.4 | 135.4 | 0.03 | 75.5 | 0.04 | 0.08 | 0.51 | 0.62 | 16.2 | 75.5 | 0.02 |
| 29 | 127.8 | 0.00 | 0.01 | 0.59 | 0.69 | 20.7 | 127.8 | 0.02 | 67.4 | 0.01 | 0.02 | 0.47 | 0.57 | 16.5 | 67.4 | 0.04 |
| 30 | 124.1 | 0.00 | 0.00 | 0.58 | 0.69 | 19.9 | 124.1 | 0.02 | 60.9 | 0.01 | 0.02 | 0.46 | 0.56 | 15.7 | 60.9 | 0.02 |
| 31 | 120.1 | 0.01 | 0.02 | 0.58 | 0.69 | 18.9 | 120.1 | 0.03 | 57.1 | 0.02 | 0.05 | 0.46 | 0.56 | 15.0 | 57.1 | 0.03 |
| 32 | 113.6 | 0.02 | 0.04 | 0.57 | 0.67 | 18.3 | 113.6 | 0.02 | 51.8 | 0.03 | 0.07 | 0.43 | 0.53 | 14.7 | 51.8 | 0.06 |
| 33 | 104 | 0.02 | 0.03 | 0.54 | 0.64 | 18.1 | 104 | 0.01 | 47.9 | 0.02 | 0.04 | 0.40 | 0.49 | 14.8 | 47.9 | 0.02 |
| 34 | 97.5 | 0.01 | 0.02 | 0.53 | 0.62 | 17.6 | 97.5 | 0.03 | 45.8 | 0.01 | 0.02 | 0.38 | 0.47 | 14.4 | 45.8 | 0.03 |
| 35 | 89 | 0.02 | 0.03 | 0.52 | 0.61 | 16.9 | 89 | 0.02 | 42 | 0.03 | 0.07 | 0.38 | 0.46 | 13.7 | 42 | 0.00 |
| 36 | 82 | 0.01 | 0.02 | 0.50 | 0.59 | 16.5 | 82 | 0.01 | 39.2 | 0.01 | 0.03 | 0.35 | 0.43 | 13.7 | 39.2 | 0.03 |
| 37 | 75.2 | 0.03 | 0.05 | 0.49 | 0.58 | 15.8 | 75.2 | 0.01 | 38.2 | 0.03 | 0.08 | 0.34 | 0.41 | 13.0 | 38.2 | 0.00 |
| 38 | 72.8 | 0.01 | 0.01 | 0.46 | 0.55 | 15.7 | 72.8 | 0.03 | 37.3 | 0.00 | 0.00 | 0.32 | 0.38 | 13.0 | 37.3 | 0.01 |
| 39 | 71.5 | 0.00 | 0.00 | 0.46 | 0.54 | 14.9 | 71.5 | 0.01 | 34.8 | 0.02 | 0.06 | 0.32 | 0.38 | 12.0 | 34.8 | 0.02 |
| 40 | 67.2 | 0.02 | 0.04 | 0.46 | 0.54 | 13.9 | 67.2 | 0.05 | 34 | 0.00 | 0.00 | 0.30 | 0.36 | 11.6 | 34 | 0.02 |
| 41 | 63.6 | 0.02 | 0.05 | 0.44 | 0.52 | 13.4 | 63.6 | 0.02 | 29.7 | 0.03 | 0.10 | 0.30 | 0.36 | 10.6 | 29.7 | 0.02 |
| 42 | 58.1 | 0.02 | 0.05 | 0.42 | 0.49 | 13.0 | 58.1 | 0.00 | 23.5 | 0.03 | 0.12 | 0.27 | 0.33 | 10.7 | 23.5 | 0.00 |
| 43 | 55.7 | 0.00 | 0.00 | 0.40 | 0.47 | 12.7 | 55.7 | 0.01 | 20.8 | 0.02 | 0.09 | 0.24 | 0.29 | 10.9 | 20.8 | 0.03 |
| 44 | 52.4 | 0.00 | 0.00 | 0.40 | 0.47 | 11.7 | 52.4 | 0.00 | 19.2 | 0.02 | 0.10 | 0.22 | 0.26 | 10.9 | 19.2 | 0.00 |
| 45 | 49.8 | 0.02 | 0.04 | 0.40 | 0.47 | 10.7 | 49.8 | 0.00 | 19 | 0.00 | 0.00 | 0.19 | 0.24 | 11.0 | 19 | 0.03 |
| 46 | 44.2 | 0.03 | 0.07 | 0.38 | 0.45 | 10.1 | 44.2 | 0.00 | 18.4 | 0.00 | 0.00 | 0.19 | 0.24 | 10.0 | 18.4 | 0.00 |
| 47 | 38.8 | 0.02 | 0.05 | 0.36 | 0.42 | 9.8 | 38.8 | 0.01 | 15 | 0.01 | 0.06 | 0.19 | 0.24 | 9.0 | 15 | 0.03 |
| 48 | 31.9 | 0.03 | 0.09 | 0.34 | 0.40 | 9.2 | 31.9 | 0.00 | 12.4 | 0.04 | 0.23 | 0.18 | 0.22 | 8.5 | 12.4 | 0.00 |
| 49 | 25 | 0.04 | 0.12 | 0.31 | 0.36 | 9.0 | 25 | 0.00 | 9.6 | 0.00 | 0.00 | 0.14 | 0.17 | 9.8 | 9.6 | 0.00 |
| 50 | 17.8 | 0.03 | 0.11 | 0.27 | 0.32 | 9.1 | 17.8 | 0.04 | 8.2 | 0.02 | 0.11 | 0.14 | 0.17 | 8.8 | 8.2 | 0.07 |
| 51 | 12.4 | 0.02 | 0.07 | 0.24 | 0.28 | 9.1 | 12.4 | 0.00 | 7.1 | 0.02 | 0.13 | 0.13 | 0.15 | 8.7 | 7.1 | 0.00 |
| 52 | 9.6 | 0.00 | 0.00 | 0.22 | 0.26 | 8.7 | 9.6 | 0.00 | 6.2 | 0.02 | 0.14 | 0.11 | 0.13 | 8.9 | 6.2 | 0.00 |
| 53 | 8.6 | 0.02 | 0.11 | 0.22 | 0.26 | 7.7 | 8.6 | 0.00 | 5.2 | 0.02 | 0.17 | 0.09 | 0.11 | 9.2 | 5.2 | 0.00 |
| 54 | 6.6 | 0.05 | 0.27 | 0.20 | 0.23 | 7.6 | 6.6 | 0.00 | 5 | 0.00 | 0.00 | 0.08 | 0.10 | 9.8 | 5 | 0.00 |
| 55 | 4.4 | 0.03 | 0.20 | 0.15 | 0.17 | 9.0 | 4.4 | 0.00 | 4 | 0.03 | 0.40 | 0.08 | 0.10 | 8.8 | 4 | 0.00 |
| 56 | 4 | 0.00 | 0.00 | 0.12 | 0.14 | 10.0 | 4 | 0.13 | 2.5 | 0.02 | 0.50 | 0.05 | 0.06 | 13.0 | 2.5 | 0.00 |
| 57 | 3.4 | 0.03 | 0.25 | 0.12 | 0.14 | 9.0 | 3.4 | 0.00 | 1 | 0.00 | 0.00 | 0.02 | 0.03 | 24.0 | 1 | 0.00 |
| 58 | 3 | 0.00 | 0.00 | 0.09 | 0.10 | 10.7 | 3 | 0.00 | 1 | 0.00 | 0.00 | 0.02 | 0.03 | 23.0 | 1 | 0.00 |
| 59 | 3 | 0.00 | 0.00 | 0.09 | 0.10 | 9.7 | 3 | 0.17 | 1 | 0.00 | 0.00 | 0.02 | 0.03 | 22.0 | 1 | 0.00 |
| 60 | 2.5 | 0.03 | 0.33 | 0.09 | 0.10 | 8.7 | 2.5 | 0.00 | 1 | 0.00 | 0.00 | 0.02 | 0.03 | 21.0 | 1 | 0.00 |
| 61 | 2 | 0.00 | 0.00 | 0.06 | 0.07 | 11.5 | 2 | 0.25 | 0.6 | 0.00 | 0.00 | 0.02 | 0.03 | 20.0 | 0.6 | 0.00 |
| 62 | 2 | 0.00 | 0.00 | 0.06 | 0.07 | 10.5 | 2 | 0.25 | 0 | 0.00 | 0.00 | 0.02 | 0.03 | 19.0 | 0 | 0.00 |
| 63 | 2 | 0.00 | 0.00 | 0.06 | 0.07 | 9.5 | 2 | 0.00 | 0 | 0.00 | 0.00 | 0.02 | 0.03 | 18.0 | 0 | 0.00 |
| 64 | 1.6 | 0.03 | 0.50 | 0.06 | 0.07 | 8.5 | 1.6 | 0.00 | 0 | 0.00 | 0.00 | 0.02 | 0.03 | 17.0 | 0 | 0.00 |
| 65 | 1 | 0.00 | 0.00 | 0.03 | 0.03 | 15.0 | 1 | 0.00 | 0 | 0.00 | 0.00 | 0.02 | 0.03 | 16.0 | 0 | 0.00 |
| 66 | 1 | 0.00 | 0.00 | 0.03 | 0.03 | 14.0 | 1 | 0.00 | 0 | 0.00 | 0.00 | 0.02 | 0.03 | 15.0 | 0 | 0.00 |
| 67 | 1 | 0.00 | 0.00 | 0.03 | 0.03 | 13.0 | 1 | 0.00 | 0 | 0.00 | 0.00 | 0.02 | 0.03 | 14.0 | 0 | 0.00 |
| 68 | 1 | 0.00 | 0.00 | 0.03 | 0.03 | 12.0 | 1 | 0.00 | 0 | 0.00 | 0.00 | 0.02 | 0.03 | 13.0 | 0 | 0.00 |
| 69 | 1 | 0.00 | 0.00 | 0.03 | 0.03 | 11.0 | 1 | 0.00 | 0 | 0.00 | 0.00 | 0.02 | 0.03 | 12.0 | 0 | 0.00 |
| 70 | 1 | 0.00 | 0.00 | 0.03 | 0.03 | 10.0 | 1 | 0.00 | 0 | 0.00 | 0.00 | 0.02 | 0.03 | 11.0 | 0 | 0.00 |
| 71 | 1 | 0.00 | 0.00 | 0.03 | 0.03 | 9.0 | 1 | 0.00 | 0 | 0.00 | 0.00 | 0.02 | 0.03 | 10.0 | 0 | 0.00 |
| 72 | 1 | 0.00 | 0.00 | 0.03 | 0.03 | 8.0 | 1 | 0.00 | 0 | 0.00 | 0.00 | 0.02 | 0.03 | 9.0 | 0 | 0.00 |
| 73 | 1 | 0.00 | 0.00 | 0.03 | 0.03 | 7.0 | 1 | 0.00 | 0 | 0.00 | 0.00 | 0.02 | 0.03 | 8.0 | 0 | 0.00 |
| 74 | 1 | 0.00 | 0.00 | 0.03 | 0.03 | 6.0 | 1 | 0.00 | 0 | 0.00 | 0.00 | 0.02 | 0.03 | 7.0 | 0 | 0.00 |
| 75 | 1 | 0.00 | 0.00 | 0.03 | 0.03 | 5.0 | 1 | 0.00 | 0 | 0.00 | 0.00 | 0.02 | 0.03 | 6.0 | 0 | 0.00 |
| 76 | 1 | 0.00 | 0.00 | 0.03 | 0.03 | 4.0 | 1 | 0.00 | 0 | 0.00 | 0.00 | 0.02 | 0.03 | 5.0 | 0 | 0.00 |
| 77 | 1 | 0.00 | 0.00 | 0.03 | 0.03 | 3.0 | 1 | 0.00 | 0 | 0.00 | 0.00 | 0.02 | 0.03 | 4.0 | 0 | 0.00 |
| 78 | 1 | 0.00 | 0.00 | 0.03 | 0.03 | 2.0 | 1 | 0.00 | 0 | 0.00 | 0.00 | 0.02 | 0.03 | 3.0 | 0 | 0.00 |
| 79 | 0 | 0.03 | 1.00 | 0.03 | 0.03 | 1.0 | 0 | 0.00 | 0 | 0.00 | 0.00 | 0.02 | 0.03 | 2.0 | 0 | 0.00 |
| 80 | 0 | 0.00 | 1.00 | 0.00 | 0.00 |  | 0 | 0.00 | 0 | 0.00 | 0.00 | 0.02 | 0.03 | 1.0 | 0 | 0.00 |
