# Supplementary material for: Comparative survival analyses among captive chimpanzees (Pan troglodytes) in America and Japan
Source: PeerJ. 2021 Aug 12;9:e11913. doi: 10.7717/peerj.11913 (PMC8364750; doi:10.7717/peerj.11913)
Supplement: Supplemental Information 3 — Please see Table S1 legend for definitions of abbreviated terms. [file peerj-09-11913-s003.docx]

|  | Females |  |  | |  |  |  | |  |  | Males |  |  |  |  |  |  |  |
| --- | --- | --- | --- | --- | --- | --- | --- | --- | --- | --- | --- | --- | --- | --- | --- | --- | --- | --- |
| Age (years) | nx (qx) | dx | qx | lx (0yr) | | lx (1yr) | | ex | nx (mx) | mx | nx (qx) | dx | qx | lx (0yr) | lx (1yr) | ex | nx (mx) | mx |
| 0 | 163.3 | 0.25 | 0.25 | | 1.00 |  | 29.5 | | 163.3 | 0.00 | 147.6 | 0.20 | 0.20 | 1.00 |  | 34.3 | 147.6 | 0.00 |
| 1 | 171.7 | 0.01 | 0.01 | | 0.75 | 1.00 | 38.1 | | 171.7 | 0.00 | 152 | 0.00 | 0.00 | 0.80 | 1.00 | 41.8 | 152 | 0.00 |
| 2 | 186.4 | 0.01 | 0.01 | | 0.74 | 0.99 | 37.5 | | 186.4 | 0.00 | 165.5 | 0.00 | 0.01 | 0.80 | 1.00 | 40.8 | 165.5 | 0.00 |
| 3 | 193.4 | 0.00 | 0.01 | | 0.73 | 0.98 | 37.0 | | 193.4 | 0.00 | 170.6 | 0.00 | 0.00 | 0.79 | 0.99 | 40.1 | 170.6 | 0.00 |
| 4 | 202.9 | 0.00 | 0.00 | | 0.73 | 0.97 | 36.1 | | 202.9 | 0.00 | 171.2 | 0.01 | 0.01 | 0.79 | 0.99 | 39.1 | 171.2 | 0.00 |
| 5 | 211.6 | 0.00 | 0.00 | | 0.73 | 0.97 | 35.1 | | 211.6 | 0.00 | 172.4 | 0.00 | 0.01 | 0.78 | 0.98 | 38.5 | 172.4 | 0.00 |
| 6 | 215.5 | 0.00 | 0.00 | | 0.73 | 0.97 | 34.1 | | 215.5 | 0.01 | 175 | 0.00 | 0.00 | 0.78 | 0.98 | 37.8 | 175 | 0.00 |
| 7 | 217.7 | 0.01 | 0.01 | | 0.73 | 0.97 | 33.1 | | 217.7 | 0.00 | 174 | 0.00 | 0.01 | 0.78 | 0.98 | 36.8 | 174 | 0.01 |
| 8 | 217.9 | 0.00 | 0.01 | | 0.72 | 0.96 | 32.4 | | 217.9 | 0.02 | 173.9 | 0.02 | 0.02 | 0.77 | 0.97 | 36.0 | 173.9 | 0.04 |
| 9 | 218.8 | 0.01 | 0.01 | | 0.72 | 0.96 | 31.6 | | 218.8 | 0.03 | 171.6 | 0.01 | 0.01 | 0.76 | 0.95 | 35.8 | 171.6 | 0.06 |
| 10 | 217.2 | 0.01 | 0.01 | | 0.71 | 0.95 | 30.9 | | 217.2 | 0.06 | 169.3 | 0.01 | 0.01 | 0.75 | 0.94 | 35.2 | 169.3 | 0.07 |
| 11 | 212.4 | 0.02 | 0.03 | | 0.70 | 0.94 | 30.1 | | 212.4 | 0.06 | 162.9 | 0.00 | 0.01 | 0.74 | 0.93 | 34.6 | 162.9 | 0.09 |
| 12 | 209.3 | 0.01 | 0.01 | | 0.68 | 0.92 | 30.0 | | 209.3 | 0.05 | 154.9 | 0.02 | 0.03 | 0.73 | 0.92 | 33.8 | 154.9 | 0.09 |
| 13 | 205 | 0.00 | 0.01 | | 0.67 | 0.90 | 29.4 | | 205 | 0.06 | 149.3 | 0.01 | 0.01 | 0.71 | 0.89 | 33.9 | 149.3 | 0.08 |
| 14 | 200.7 | 0.01 | 0.01 | | 0.67 | 0.90 | 28.5 | | 200.7 | 0.06 | 145.1 | 0.00 | 0.01 | 0.70 | 0.88 | 33.4 | 145.1 | 0.08 |
| 15 | 196.8 | 0.01 | 0.01 | | 0.66 | 0.89 | 27.8 | | 196.8 | 0.05 | 142.8 | 0.00 | 0.01 | 0.70 | 0.87 | 32.6 | 142.8 | 0.05 |
| 16 | 194.6 | 0.01 | 0.01 | | 0.66 | 0.88 | 27.1 | | 194.6 | 0.05 | 141.8 | 0.00 | 0.01 | 0.69 | 0.87 | 31.8 | 141.8 | 0.07 |
| 17 | 188.4 | 0.01 | 0.01 | | 0.65 | 0.87 | 26.3 | | 188.4 | 0.08 | 141.4 | 0.00 | 0.00 | 0.69 | 0.86 | 31.0 | 141.4 | 0.05 |
| 18 | 180.8 | 0.00 | 0.01 | | 0.64 | 0.86 | 25.6 | | 180.8 | 0.03 | 138 | 0.01 | 0.02 | 0.69 | 0.86 | 30.0 | 138 | 0.06 |
| 19 | 175.4 | 0.01 | 0.01 | | 0.64 | 0.86 | 24.8 | | 175.4 | 0.05 | 130.7 | 0.03 | 0.05 | 0.67 | 0.84 | 29.7 | 130.7 | 0.08 |
| 20 | 168.1 | 0.02 | 0.03 | | 0.63 | 0.85 | 24.0 | | 168.1 | 0.04 | 123 | 0.02 | 0.02 | 0.64 | 0.80 | 30.0 | 123 | 0.09 |
| 21 | 158.9 | 0.01 | 0.01 | | 0.61 | 0.82 | 23.7 | | 158.9 | 0.04 | 120 | 0.00 | 0.00 | 0.63 | 0.79 | 29.7 | 120 | 0.04 |
| 22 | 154.2 | 0.00 | 0.01 | | 0.61 | 0.81 | 23.0 | | 154.2 | 0.04 | 117.8 | 0.02 | 0.03 | 0.63 | 0.79 | 28.7 | 117.8 | 0.04 |
| 23 | 151.8 | 0.01 | 0.02 | | 0.60 | 0.81 | 22.2 | | 151.8 | 0.04 | 113 | 0.01 | 0.02 | 0.61 | 0.77 | 28.4 | 113 | 0.03 |
| 24 | 147.2 | 0.00 | 0.01 | | 0.59 | 0.79 | 21.6 | | 147.2 | 0.03 | 104 | 0.03 | 0.06 | 0.60 | 0.75 | 27.9 | 104 | 0.03 |
| 25 | 140.4 | 0.01 | 0.01 | | 0.59 | 0.79 | 20.7 | | 140.4 | 0.03 | 93.5 | 0.02 | 0.03 | 0.57 | 0.71 | 28.6 | 93.5 | 0.04 |
| 26 | 137.8 | 0.01 | 0.02 | | 0.58 | 0.77 | 20.0 | | 137.8 | 0.03 | 90.5 | 0.00 | 0.00 | 0.55 | 0.69 | 28.4 | 90.5 | 0.02 |
| 27 | 128.1 | 0.02 | 0.04 | | 0.57 | 0.76 | 19.3 | | 128.1 | 0.01 | 82.2 | 0.01 | 0.02 | 0.55 | 0.69 | 27.4 | 82.2 | 0.04 |
| 28 | 119.8 | 0.00 | 0.01 | | 0.55 | 0.73 | 19.0 | | 119.8 | 0.03 | 73.4 | 0.02 | 0.04 | 0.53 | 0.67 | 27.1 | 73.4 | 0.07 |
| 29 | 109.2 | 0.00 | 0.01 | | 0.54 | 0.73 | 18.2 | | 109.2 | 0.02 | 66.8 | 0.01 | 0.01 | 0.51 | 0.64 | 27.2 | 66.8 | 0.05 |
| 30 | 101.6 | 0.02 | 0.03 | | 0.54 | 0.72 | 17.3 | | 101.6 | 0.03 | 61.5 | 0.00 | 0.00 | 0.51 | 0.63 | 26.6 | 61.5 | 0.03 |
| 31 | 95.4 | 0.02 | 0.03 | | 0.52 | 0.70 | 16.8 | | 95.4 | 0.01 | 59.4 | 0.01 | 0.02 | 0.51 | 0.63 | 25.6 | 59.4 | 0.03 |
| 32 | 87.5 | 0.02 | 0.03 | | 0.51 | 0.68 | 16.3 | | 87.5 | 0.06 | 55.7 | 0.01 | 0.02 | 0.50 | 0.62 | 25.0 | 55.7 | 0.08 |
| 33 | 85 | 0.00 | 0.00 | | 0.49 | 0.66 | 15.9 | | 85 | 0.04 | 51 | 0.00 | 0.00 | 0.49 | 0.61 | 24.4 | 51 | 0.04 |
| 34 | 81.5 | 0.02 | 0.05 | | 0.49 | 0.66 | 14.9 | | 81.5 | 0.02 | 48.3 | 0.03 | 0.06 | 0.49 | 0.61 | 23.4 | 48.3 | 0.03 |
| 35 | 78.5 | 0.00 | 0.00 | | 0.47 | 0.62 | 14.6 | | 78.5 | 0.02 | 43 | 0.04 | 0.09 | 0.46 | 0.58 | 23.8 | 43 | 0.05 |
| 36 | 75.2 | 0.01 | 0.03 | | 0.47 | 0.62 | 13.6 | | 75.2 | 0.03 | 39.2 | 0.00 | 0.00 | 0.42 | 0.53 | 25.1 | 39.2 | 0.03 |
| 37 | 66.1 | 0.01 | 0.01 | | 0.45 | 0.61 | 12.9 | | 66.1 | 0.04 | 34.8 | 0.04 | 0.09 | 0.42 | 0.53 | 24.1 | 34.8 | 0.01 |
| 38 | 61.2 | 0.01 | 0.03 | | 0.45 | 0.60 | 12.1 | | 61.2 | 0.03 | 27.3 | 0.02 | 0.07 | 0.38 | 0.48 | 25.2 | 27.3 | 0.04 |
| 39 | 56.7 | 0.01 | 0.02 | | 0.43 | 0.58 | 11.4 | | 56.7 | 0.04 | 23.6 | 0.00 | 0.00 | 0.36 | 0.45 | 25.9 | 23.6 | 0.02 |
| 40 | 50.2 | 0.02 | 0.04 | | 0.43 | 0.57 | 10.6 | | 50.2 | 0.01 | 22.2 | 0.00 | 0.00 | 0.36 | 0.45 | 24.9 | 22.2 | 0.02 |
| 41 | 43 | 0.03 | 0.07 | | 0.41 | 0.55 | 10.0 | | 43 | 0.01 | 17.9 | 0.02 | 0.05 | 0.36 | 0.45 | 23.9 | 17.9 | 0.05 |
| 42 | 34.6 | 0.03 | 0.08 | | 0.38 | 0.51 | 9.7 | | 34.6 | 0.00 | 13.6 | 0.00 | 0.00 | 0.34 | 0.43 | 24.1 | 13.6 | 0.04 |
| 43 | 26.4 | 0.01 | 0.04 | | 0.35 | 0.47 | 9.5 | | 26.4 | 0.00 | 12.6 | 0.00 | 0.00 | 0.34 | 0.43 | 23.1 | 12.6 | 0.04 |
| 44 | 21.8 | 0.02 | 0.05 | | 0.33 | 0.45 | 8.8 | | 21.8 | 0.00 | 12 | 0.00 | 0.00 | 0.34 | 0.43 | 22.1 | 12 | 0.04 |
| 45 | 18.3 | 0.02 | 0.06 | | 0.32 | 0.43 | 8.2 | | 18.3 | 0.00 | 10.9 | 0.00 | 0.00 | 0.34 | 0.43 | 21.1 | 10.9 | 0.00 |
| 46 | 15.9 | 0.02 | 0.07 | | 0.30 | 0.40 | 7.7 | | 15.9 | 0.00 | 8.6 | 0.00 | 0.00 | 0.34 | 0.43 | 20.1 | 8.6 | 0.06 |
| 47 | 12.5 | 0.04 | 0.14 | | 0.28 | 0.37 | 7.2 | | 12.5 | 0.00 | 8 | 0.00 | 0.00 | 0.34 | 0.43 | 19.1 | 8 | 0.00 |
| 48 | 10.6 | 0.00 | 0.00 | | 0.24 | 0.32 | 7.2 | | 10.6 | 0.00 | 5.7 | 0.05 | 0.14 | 0.34 | 0.43 | 18.1 | 5.7 | 0.09 |
| 49 | 7.7 | 0.03 | 0.11 | | 0.24 | 0.32 | 6.2 | | 7.7 | 0.00 | 4.6 | 0.00 | 0.00 | 0.29 | 0.37 | 20.0 | 4.6 | 0.00 |
| 50 | 5.9 | 0.04 | 0.17 | | 0.21 | 0.29 | 5.8 | | 5.9 | 0.00 | 4 | 0.00 | 0.00 | 0.29 | 0.37 | 19.0 | 4 | 0.00 |
| 51 | 4.1 | 0.04 | 0.20 | | 0.18 | 0.24 | 5.8 | | 4.1 | 0.00 | 3.5 | 0.00 | 0.00 | 0.29 | 0.37 | 18.0 | 3.5 | 0.00 |
| 52 | 3.6 | 0.00 | 0.00 | | 0.14 | 0.19 | 6.0 | | 3.6 | 0.00 | 3 | 0.00 | 0.00 | 0.29 | 0.37 | 17.0 | 3 | 0.00 |
| 53 | 2.6 | 0.05 | 0.33 | | 0.14 | 0.19 | 5.0 | | 2.6 | 0.00 | 2.6 | 0.00 | 0.00 | 0.29 | 0.37 | 16.0 | 2.6 | 0.00 |
| 54 | 1.6 | 0.00 | 0.00 | | 0.09 | 0.13 | 6.0 | | 1.6 | 0.00 | 2 | 0.00 | 0.00 | 0.29 | 0.37 | 15.0 | 2 | 0.00 |
| 55 | 1 | 0.00 | 0.00 | | 0.09 | 0.13 | 5.0 | | 1 | 0.00 | 2 | 0.00 | 0.00 | 0.29 | 0.37 | 14.0 | 2 | 0.00 |
| 56 | 1 | 0.00 | 0.00 | | 0.09 | 0.13 | 4.0 | | 1 | 0.00 | 1.6 | 0.00 | 0.00 | 0.29 | 0.37 | 13.0 | 1.6 | 0.00 |
| 57 | 1 | 0.00 | 0.00 | | 0.09 | 0.13 | 3.0 | | 1 | 0.00 | 1 | 0.00 | 0.00 | 0.29 | 0.37 | 12.0 | 1 | 0.00 |
| 58 | 1 | 0.00 | 0.00 | | 0.09 | 0.13 | 2.0 | | 1 | 0.00 | 1 | 0.00 | 0.00 | 0.29 | 0.37 | 11.0 | 1 | 0.00 |
| 59 | 0.1 | 0.09 | 1.00 | | 0.09 | 0.13 | 1.0 | | 0.1 | 0.00 | 1 | 0.00 | 0.00 | 0.29 | 0.37 | 10.0 | 1 | 0.00 |
| 60 | 0 | 0.00 | 1.00 | | 0.00 | 0.00 |  | | 0 | 0.00 | 1 | 0.00 | 0.00 | 0.29 | 0.37 | 9.0 | 1 | 0.00 |
| 61 | 0 | 0.00 | 1.00 | | 0.00 | 0.00 |  | | 0 | 0.00 | 1 | 0.00 | 0.00 | 0.29 | 0.37 | 8.0 | 1 | 0.00 |
| 62 | 0 | 0.00 | 1.00 | | 0.00 | 0.00 |  | | 0 | 0.00 | 1 | 0.00 | 0.00 | 0.29 | 0.37 | 7.0 | 1 | 0.00 |
| 63 | 0 | 0.00 | 1.00 | | 0.00 | 0.00 |  | | 0 | 0.00 | 1 | 0.00 | 0.00 | 0.29 | 0.37 | 6.0 | 1 | 0.00 |
| 64 | 0 | 0.00 | 1.00 | | 0.00 | 0.00 |  | | 0 | 0.00 | 1 | 0.00 | 0.00 | 0.29 | 0.37 | 5.0 | 1 | 0.00 |
| 65 | 0 | 0.00 | 1.00 | | 0.00 | 0.00 |  | | 0 | 0.00 | 1 | 0.00 | 0.00 | 0.29 | 0.37 | 4.0 | 1 | 0.00 |
| 66 | 0 | 0.00 | 1.00 | | 0.00 | 0.00 |  | | 0 | 0.00 | 1 | 0.00 | 0.00 | 0.29 | 0.37 | 3.0 | 1 | 0.00 |
| 67 | 0 | 0.00 | 1.00 | | 0.00 | 0.00 |  | | 0 | 0.00 | 1 | 0.00 | 0.00 | 0.29 | 0.37 | 2.0 | 1 | 0.00 |
| 68 | 0 | 0.00 | 1.00 | | 0.00 | 0.00 |  | | 0 | 0.00 | 0 | 0.29 | 1.00 | 0.29 | 0.37 | 1.0 | 0 | 0.00 |
| 69 | 0 | 0.00 | 1.00 | | 0.00 | 0.00 |  | | 0 | 0.00 | 0 | 0.00 | 1.00 | 0.00 | 0.00 |  | 0 | 0.00 |
